# A novel modifier gene therapy to treat Stargardt disease: Phase 1 GARDian1 Trial Insights

**DOI:** 10.1038/s41433-025-04202-5

**Published:** 2026-01-10

**Authors:** Arshad M. Khanani, Lejla Vajzovic, Benjamin Bakall, Venkata Ramana Murthy Chavali, Huma Qamar

**Affiliations:** 1 Sierra Eye Associates, Reno, NV USA; 2 University of Nevada, Reno School of Medicine, Reno, NV USA; 3 Duke University, Durham, NC USA; 4 Associated Retina Consultants, Phoenix, AZ USA; 5 University of Arizona, College of Medicine-Phoenix, Phoenix, AZ USA; 6 Ocugen, Inc., Malvern, PA USA

**Keywords:** Retina, Vision disorders

## Introduction

Stargardt disease type 1 (STGD1) is an orphan inherited macular dystrophy affecting over 100,000 individuals in the US and EU, commonly presenting in childhood or adolescence [1]. Biallelic *ABCA4* mutations lead to toxic bisretinoid lipofuscin accumulation, retinal pigment epithelium (RPE) atrophy, and progressive central vision loss [2]. With no approved treatments, STGD1 remains a significant unmet medical need. OCU410ST (AAV5-hRORA) is designed to modulate RPE and photoreceptor homeostasis, thereby preserving retinal integrity independent of *ABCA4* genotype. This first-in-human phase 1 trial (NCT05956626) evaluated safety, tolerability, and exploratory efficacy of a single subretinal dose of OCU410ST in adults with advanced STGD1.

## Methods

GARDian1 was a multicentre, open-label, 3 + 3 dose escalation study. Three patients per cohort received a single 200 µl subretinal injection of OCU410ST (low: 3.75 × 10^10^ vg/mL; medium: 7.5 × 10^10^ vg/mL; or high: 2.25 × 10^11^ vg/mL) in the worse seeing study eye. Key eligibility criteria included age 18–65 years, biallelic *ABCA4* variants, early to advanced bull’s eye maculopathy, best corrected visual acuity (BCVA) ≥ 50 ETDRS letters, and preserved outer nuclear layer. Follow-up visits occurred at baseline and months 1, 3, 6, 9, and 12. The primary objective was to assess the safety and tolerability of OCU410ST, including treatment-related safety adverse events (AEs). Exploratory endpoints included change from baseline to Month 12 in the progression of definitely decreased autofluorescence (DDAF) lesion area (treated vs untreated fellow eyes), reported as absolute area (mm^2^) and square root–transformed area (√DDAF, mm) to account for baseline lesion size, and BCVA. Additional structural and functional measures were collected but are not reported here. Analyses were descriptive, with paired methods used for between-eye comparisons when appropriate.

## Results

Nine patients with STGD1 (5 Females/4 Males; mean age 41.7 ± 18.1 years) received OCU410ST in the study eye. Eight of nine patients (89%) completed the 12-month visit; one high-dose patient was lost to follow-up. Treatment and surgery were well tolerated with no drug-related serious AEs or adverse events of special interest. Treatment-emergent AEs (TEAEs) unrelated to OCU410ST occurred in 8 of 9 patients (89%), totalling 30 events (73% Grade 1, 27% Grade 2).

**Fig. 1:**
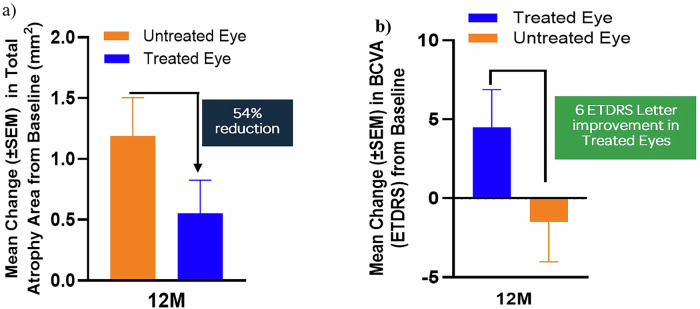
Change in Atrophic Lesion Growth and Visual Acuity at M12 following OCU410ST Treatment. Treated eyes showed a decrease in atrophic lesion growth from baseline (**a**), and stabilisation/improvement in Visual Acuity (**b**).

Efficacy was assessed in predefined evaluable subsets (Supplementary Table 1). Among six patients with gradable Fundus Auto Fluorescence images, mean atrophic lesion growth was reduced by 54%: treated eyes progressed 0.55 ± 0.27 mm^2^ versus 1.19 ± 0.31 mm^2^ in untreated eyes (Fig. 1a). Annual √DDAF expansion was 0.10 ± 0.039 mm/year in treated eyes (50% lesion reduction) versus 0.19 ± 0.026 mm/year in fellow eyes. The rate in treated eyes was below published natural history rates (0.14–0.18 mm/year). Among six BCVA-evaluable patients without confounders, treated eyes improved by +4.5 ± 2.20 ETDRS letters at Month 12, compared with −1.5 ± 2.33 letters in untreated fellow eyes, yielding a +6-letter gain (Fig. 1b). All treated eyes either stabilised (±4 letters) or improved (≥5 letters) in visual acuity.

## Discussion

OCU410ST demonstrated a favourable safety profile. TEAEs were mild/moderate and attributable to procedural effects, underlying disease progression, or comorbidities.

Slowing of DDAF progression (0.10 mm/year √DDAF) was below published natural history rates (0.14–0.18 mm/year), a validated surrogate endpoint for RPE and photoreceptor loss that predicts functional decline in STGD1 [3]. BCVA gains in treated eyes counteract the expected natural history of decline (0.5–2.8 ETDRS letters/year overall; ~2 letters/year in advanced disease), where ≥5-letter change correlates with clinically meaningful reading and face recognition improvements. BCVA stabilisation or gain preserves central vision, critical for quality of life [4]. No globally approved therapies for STGD1 exist. Full *ABCA4* replacement remains limited by viral vector packaging capacity, while other alternative strategies, such as dual-vector approaches, CRISPR, and RNA-targeting, are still in early development and face important safety and efficacy hurdles. Emerging data from Belite Bio’s Tinlarebant, the only other pivotal STGD programme, showed no visual function gains, limited 2-year lesion reduction in adolescents, and safety concerns [5]. In contrast, the convergent functional and structural benefits, in addition to safety, underscore the novelty of the RORA-mediated neuroprotection approach, offering a paradigm shift from genotype-constrained replacement to agnostic modification [5]. GARDian1 trial achieved its primary objective of favourable safety and provides confirmatory evidence of functional and structural benefit, demonstrating the potential of OCU410ST to slow macular atrophic progression and transform the treatment landscape for patients with significant unmet need.

### Data sharing

Data from GARDian1 will be available upon reasonable request. Trial registration: NCT05956626.

## Supplementary information

Supplementary Material
