# Supplementary material for: A novel modifier gene therapy to treat Stargardt disease: Phase 1 GARDian1 Trial Insights
Source: Eye (Lond). 2026 Jan 10;40(3):429–30. doi: 10.1038/s41433-025-04202-5 (PMC12881397; doi:10.1038/s41433-025-04202-5)
Supplement: Supplementary file 1 — Supplementary Material [file 41433_2025_4202_MOESM1_ESM.docx]

**Title: A Novel Modifier Gene Therapy to Treat Stargardt Disease: Phase 1 GARDian1 Insights**

Authors: Arshad M. Khanani MD, MA, FASRS^1,2^, Lejla Vajzovic, MD, FASRS^3^, Benjamin Bakall, MD, PhD^4,5^, Venkata Ramana Murthy Chavali, PhD^6^ and Huma Qamar, MD, MPH, CMI^6^

Supplementary Table S1. OCU410ST Stabilizes or Improves Retinal Structure and Function

| Subject | C1P01 | C1P02 | C1P03 | C2P01 | C2P02 | C2P03 | C3P01 | C3P02 | C3P03 |
| --- | --- | --- | --- | --- | --- | --- | --- | --- | --- |
| Dose | Low | Low | Low | Med | Med | Med | High | High | High |
| Atrophic lesion growth (mm^2^) compared to untreated eyes | ✓ | ✓ | ✓ | & | # | ✓ | - | LTFU | ✓ |
| Visual Function (BCVA) compared to untreated eye | * | ✓ | ✓ | * | ✓ | ✓ | ✓ | LTFU | ✓ |

BCVA=best-corrected visual acuity; LTFU=loss to follow-up

✓= Parameters showing stabilization/improvement in treated eye or when compared to untreated fellow eye

*Subjects not included in assessment due to cataract, C3P02 is loss to follow-up, C3P01 had partial foveal detachment during surgery which did not attach

& Subject with indeterminate lesion size due to advanced cataract for this patient

^#^Subjects that did not show improvement compared to untreated fellow eyes

For BCVA, Stabilization = ±4 ETDRS Letters from baseline or improvement when compared to untreated eyes; Improvement =≥5 ETDRS letters from baseline or improvement when compared to untreated fellow eyes

For Atrophic Lesion growth, Stabilization = Mean change from baseline in lesion growth is similar to that of the fellow eye; Improvement= Mean change from baseline in lesion growth is lesser when compared to the fellow eye.

**Supplementary Table 2.** OCU410ST Study Group. Study teams who participated at each study site in the Phase 1 trial.

| Name | Location | Principal Investigator |
| --- | --- | --- |
| Associated Retina Consultants | Phoenix, AZ | Benjamin Bakall (I), Jillian Bollinger (C), Mallory Mintert (C) |
| Duke Eye Center | Durhan, NC | Ramiro Maldonado (I), Lyndsay Williamson (C) |
| Retina Foundation of Southwest | Dallas, TX | Karl Csaky (I), Rebecca Tiang (C) |
| Retina Consultants of Texas | Bellaire, TX | Charles Wykoff (I), Rebecca Tiang © |
| Bascom Palmer Eye Institute | Miami, FL | Byron L. Lam (I), Ninel Z. Gregori (SI), Adriana Drada (C), Brandon Sparling (OT), Osmany R. Gil Figueredo (OT), Liu Mu (OT) |
| Mississippi Retina Associates | Jackson, MI | Michael J Borne (I), Anne Britt (C), Mallie Taylor (C) |
| I = Investigator; C = Coordinator; OT = Ophthalmic Technician; Ph = Pharmacist | | |
